# Order from chaos: Using CSF proteomics to predict ALS progression

**DOI:** 10.1002/acn3.51910

**Published:** 2023-09-27

**Authors:** Xiuli Yang, Lindsey R. Hayes

**Affiliations:** ^1^ Department of Neurology Brain Science Institute, Johns Hopkins University Baltimore Maryland 21205 USA

Amyotrophic lateral sclerosis (ALS) carries a uniformly poor prognosis with a median survival of 2–5 years after onset.^1^ However, individual patient disease trajectories vary widely.^2^ Variability in the rate of ALS progression remains poorly understood, poses a major hurdle for clinical trial design and interpretation, and limits clinicians' ability to counsel patients. Moreover, clinical progression, as commonly assessed by the Revised ALS Functional Rating Scale (ALSFRS‐R), does not occur in a linear manner as once assumed.^2^, ^3^ There is an unmet need for biomarkers of ALS disease activity and progression, which Vu and colleagues seek to address in the current issue.^4^

The ALS field has been constrained by a lack of effective biomarkers, including diagnostic, prognostic, and pharmacodynamic tools, but that landscape is rapidly evolving.^5^ Arguably the best‐established prognostic biomarker in ALS is neurofilament light chain (NfL), a neuron‐specific marker of axonal damage.^6^ Blood and CSF levels of NfL and other neurofilament subunits are elevated in ALS, often to a greater degree than in other neurodegenerative and neuroinflammatory disorders, though the lack of a well‐defined cutoff limits diagnostic utility.^7^ Longitudinal studies in ALS mutation carriers show a presymptomatic rise in NfL that continues to increase for approximately a year before plateauing.^8^ Higher initial NfL levels therefore predict more rapid progression,^9^ but NfL stabilization over time prevents its use as a marker of ongoing disease activity. NfL is not yet widely utilized in clinical practice but has become a key marker of efficacy in clinical trials, spurred by the recent FDA approval of tofersen based on NfL lowering in blood.^10^ Other candidate CSF markers of disease progression include inflammatory markers such as chitotriosidase 1 (CHIT1)^11^, ^12^ and CSF cytokine levels.^13^ However, none outperformed NfL in head‐to‐head comparison. Finally, two preliminary reports detail the discovery of a novel class of CSF biomarkers, comprised of cryptic peptides arising from TDP‐43 loss of function.^14^, ^15^ CSF cryptic peptides offer an exciting mechanistic readout for TDP‐43 proteinopathy, a major pathologic hallmark of ALS, though their utility as clinical biomarkers remains to be fully explored. An initial longitudinal analysis of the HDGFL2 cryptic peptide shows that levels rise presymptomatically and stabilize or fall over time.^14^ Thus, there is a continued need for biomarkers of ALS disease activity and progression over the full disease trajectory.

In the current study, Vu and colleagues report the results of unbiased proteomics analysis on longitudinal cerebrospinal fluid (CSF) samples from fast versus slow progressing patients and a mathematical model for the prediction of ALS progression rate.^4^ Shotgun proteomics was performed on longitudinal CSF samples from a discovery cohort of 11 ALS patients, separated into fast versus slow progressors by the rate of change of the ALSFRS‐R score. Numerous differentially expressed proteins were identified based on rate of progression, including NfL and CHIT1. Fast progressors showed upregulation of inflammatory signaling pathways and downregulated synaptogenesis and metabolic pathways. Multivariate analysis of 59 proteins that consistently separated patients by rate of progression identified three markers, retinoid binding protein 4 (RBP4), kallistatin (SERPINA4), and coagulation factor XII (F12) that reliably distinguished fast versus slow progressors with improved sensitivity and specificity over any single marker. RBP4, SERPINA4, and F12 were next analyzed by ELISA in the discovery cohort and a validation cohort of 11 patients. In the validation cohort, SERPINA4 alone was significantly increased in fast progressors and performed as well as the combination of all three markers. Finally, based on the recognition that overall proteome variance was increased in fast versus slow progressing patients, a mathematical model was generated utilizing the CSF proteome variance or ‘entropy’ as a marker of ALS progression. The resulting state‐transition model, based on principal component analysis of the mass spectrometry data, was used to simulate the progressive deregulation of the CSF proteome from the time of onset to ALS diagnosis. Interestingly, this analysis identified a slow‐progressing patient with increasing CSF proteome variance over time, suggesting the potential ability to detect acceleration of disease progression.

Limitations of the study include the modest number of patients, restricted by the resource‐intensive nature of discovery proteomics as well as the availability of longitudinal CSF samples. Because NfL was not among the most stringent list of 59 markers in the discovery cohort, the potential contribution of NfL to the sensitivity and specificity of the panel remains to be determined. Interestingly, RBP4, SERPINA4, and F12 are not CNS‐specific proteins, and thus the central versus peripheral origin of the increased CSF levels remains uncertain. Future analysis in paired plasma/CSF sample sets is needed to further clarify the potential pathophysiologic significance of these markers.

Perhaps the most intriguing aspect of the study is the mathematical model, which raises the possibility that overall dysregulation of CSF protein networks may better predict ALS progression than changes in individual CSF markers. The feasibility of translation of such an approach to the clinic is uncertain, and “CSF entropy” remains to be compared to NfL as the reigning gold standard. However, it is tempting to consider chaos itself as a fitting benchmark for the formidable clinical heterogeneity of ALS.

## Conflicts of Interest

The authors declare no competing interests.
